# Follow-Up Comparison of Fluorescence Optical Imaging With Musculoskeletal Ultrasound for Early Detection of Psoriatic Arthritis

**DOI:** 10.3389/fmed.2022.845545

**Published:** 2022-03-18

**Authors:** Juliane Büttner, Anne-Marie Glimm, Georgios Kokolakis, Magdalena Erdmann-Keding, Gerd-Rüdiger Burmester, Paula Hoff, Jens Klotsche, Sarah Ohrndorf

**Affiliations:** ^1^Department of Rheumatology and Clinical Immunology, Charité – Universitätsmedizin Berlin, Berlin, Germany; ^2^Department of Endocrinology, Nephrology and Rheumatology, Universitätsklinikum Leipzig, Leipzig, Germany; ^3^Department of Dermatology, Venereology and Allergology, Charité – Universitätsmedizin Berlin, Berlin, Germany; ^4^Haut- & Lasercentrum, Dr. Tanja Fischer und Kollegen, Potsdam – Berlin, Berlin, Germany; ^5^Endokrinologikum Berlin am Gendarmenmarkt, Berlin, Germany; ^6^German Rheumatism Research Centre Berlin (DRFZ), Leibniz Association, Berlin, Germany; ^7^Institute for Social Medicine, Epidemiology and Health Economics, Charité – Universitätsmedizin Berlin, Berlin, Germany

**Keywords:** psoriatic arthritis, fluorescence optical imaging, ultrasound imaging, follow-up studies (MeSH), hand, inflammation

## Abstract

**Objectives:**

Early diagnosis of psoriatic arthritis (PsA) is crucial for a patient outcome but hampered by heterogenous manifestation and a lack of specific biomarkers. We recently showed that fluorescence optical imaging (FOI) can differentiate between patients with confirmed and suspected PsA. This study aims to follow-up (FU) patients with confirmed and suspected PsA focusing on patients with a change from suspected to confirmed PsA by the use of FOI in comparison with musculoskeletal ultrasound (MSUS).

**Methods:**

Follow-up examination of patients included in the study performed by Erdmann-Keding et al. in which FOI of both hands was performed in a standardized manner using three predefined phases (p1–p3) and PrimaVista Mode (PVM). The comparison was drawn to grayscale–power Doppler (GS/PD) MSUS of the clinically dominant hand (wrist, MCP, PIP, DIP 2–5) from dorsal or palmar.

**Results:**

Patients with a change from suspected to diagnosed PsA showed an increased prevalence of joints with pathological enhancement in FOI (*p* = 0.046) with an unchanged joint distribution pattern, especially with a dominant involvement of DIP joints. Compared to the baseline, these patients were three times more common to show enhancement in FOI p3 at FU. Newly detected pathologic joints by FOI (PVM, p2) and MSUS at FU were positively associated with the change of diagnosis from suspected to confirmed PsA (FOI: AUC 0.78; GSUS: AUC 0.77).

**Conclusion:**

Fluorescence optical imaging appears to be a helpful tool to detect early PsA and to distinguish between acute and chronic disease stages. It could thereby become a suitable tool as a screening method to select psoriasis patients with an indication for further rheumatological evaluation.

## Introduction

Psoriatic arthritis (PsA) affects up to 30% of patients suffering from psoriasis (1, 2) and is characterized by systemic inflammation and extensive synovitis, which results in erosions of articular cartilage and bone that leads to joint destruction (3). As PsA is destructive and progressive as rheumatoid arthritis (RA), delay in diagnosis of 6 and 12 months impacts long-term joint damage and functional disability (4, 5). About 20% of the patients develop very destructive disabling arthritis (6). Hence, not only an early diagnosis of PsA but also methods to identify “at risk” patients for developing PsA are decisive.

Nonetheless, diagnosing PsA remains challenging since there are no specific biomarkers (7).

In addition, ClASsification criteria for Psoriatic Arthritis (CASPAR) was found to have exceptional specificity for PsA but is inappropriate to screen patients with PsO for PsA development due to low sensitivity (8). Several imaging modalities are used in the diagnostic process for PsA that includes magnetic resonance imaging (MRI) and musculoskeletal ultrasound (MSUS) in grayscale (GS) and in power Doppler (PD) mode. FOI with the Xiralite^®^ system is a novel technology, which is sensitive for detecting inflammatory joint processes of the hands. It uses near-infrared light to visualize altered microcirculation such as hyperperfusion, neoangiogenesis, and capillary leakage after the application of a fluorescence dye (9).

The previous studies have already provided evidence that FOI is suitable for therapy monitoring in early rheumatoid or inflammatory arthritis (10, 11) and also for the detection of inflammatory skin changes in the hands and PsA-typical signal patterns (12, 13).

The high prevalence (10.1–15.5%) of undiagnosed PsA in patients with psoriasis requires a sensitive screening tool to select patients with an indication for further rheumatological evaluation (14). Erdmann-Keding et al. compared FOI and MSUS in detecting joint inflammation in patients with confirmed and suspected PsA (15). In this study, we performed follow-up (FU) examinations of the same cohort of patients to further investigate signal enhancement in different FOI phases (p1–p3), which represents acute or chronic inflammation.

The aim of this study was to explore the potential of FOI for making an early diagnosis of PsA concentrating on those patients who underwent a change of diagnosis from suspected to diagnosed PsA between the two studies and to examine the possible application of FOI as a screening tool to identify exactly these patients.

## Materials and Methods

The study was conducted as a cooperation between the departments of Dermatology and Rheumatology of the Charité – Universitätsmedizin Berlin, Germany – and approved by its local ethic committee (EA1/025/10). Patients were included after written informed consent to participate in the study.

The study was designed as FU study to Erdmann-Keding et al. (11) which compared FOI with MSUS and clinical examination (CE) in 60 patients suffering from confirmed (*n* = 26) or suspected PsA (*n* = 34).

Patients were contacted between May 2014 and January 2015 *via* post or telephone to participate in the present FU study. Baseline data were collected between March 2010 to November 2011 and FU data between May 2014 and January 2015.

The recruited patients were assigned to three different groups:

• Diagnosed PsA after baseline assessment (group I).
• Still suspected PsA (group II).
• (Unchanged) Diagnosed PsA (group III).

The diagnosis of PsA was confirmed by the treating dermatologist or rheumatologist based on the medical history and clinical evaluation before the FU examination (Supplementary Figure 1).

To ensure good comparability to the baseline (BL) data from 2011, patients underwent the same assessments including a CE, MSUS in GS/PD of the clinically dominant hand (wrist, MCP, PIP, DIP), and FOI of both hands.

### Clinical Examination

For CE, the Disease Activity Score 28 (DAS28) was used (16).

Skin involvement was evaluated by body surface area (BSA), Nail Psoriasis Severity Index (NAPSI), and the Psoriasis Area Severity Index (PASI). A visual analog scale (VAS 0–10 mm) was used to examine the patient’s global assessment of joint pain, skin involvement, and pruritus (17–19).

### Musculoskeletal Ultrasound

Musculoskeletal ultrasound examination (Esaote Mylab Twice, Genova; Italy) of the clinically dominant hand was performed by grayscale (GS) and power Doppler (PD) MSUS from dorsal and palmar using a linear transducer with 10–18 MHz. GSUS and PDUS were performed by following the EULAR recommendations and OMERACT definitions (20, 21).

To avoid a possible variance between different sonography devices and examiners, all patients were examined on the same ultrasound machine and examiner (SO) at BL and FU.

The sonographer is a EULAR-certified Teacher (Level II) with a relatively long ultrasound experience of about 10 years at the time of FU examination.

Settings for PDUS were as follows: pulse repetition frequency 0.75 kHz, power Doppler frequency 11.1 MHz, wall filter 3.

The wrist, metacarpophalangeal (MCP) joints 2–5, proximal interphalangeal joints (PIP) 2–5, and distal interphalangeal joints (DIP) 2–5 were evaluated semiquantitatively for synovitis [0 = absent, 1 = mild, 2 = moderate, 3 = severe; (22, 23)] and for tenosynovitis (0–1) in both GSUS and PDUS modes. Superficial erosions were scored for the presence and absence (0–1). For each patient, a sum score of all joints was calculated.

### Fluorescence Optical Imaging

Fluorescence optical imaging (FOI) was performed with the Xiralite X4 device (Xiralite GmbH, Berlin, Germany) following a standardized procedure. The total examination time lasted 360 s including intravenously administration of indocyanine green (ICG) bolus (ICG-Pulsion, 0.1 mg/kg/body weight) 10 s after the beginning. By recording one image per second, the system provided 360 images in total. Alteration of the dye concentrations as signal intensity was presented by false color scale. For evaluation, a film modus with three predefined phases based on signal intensity in the fingertips (p1–p3) and an automatically generated composite image (PrimaVista Mode, PVM) were considered.

Phase 1 refers to the period between the start of the examination, the injection of ICG, and the beginning of the increased signal intensity in the fingertips whereas phase 2 includes remaining increased signal intensities in the fingertips recognizable by the red color. Phase 3 is defined by missing high signals in the fingertips until the end of the image stack (9, 12, 24).

To analyze joint activity, a semiquantitative grading system for wrist, MCP 2–5, PIP 2–5, and DIP 2–5 of each hand from grade 0 to 3 [0 = no signal enhancement, 1 = low signal enhancement ≤ 25%, 2 = moderate signal enhancement (>25%, ≤50%), 3 = strong signal enhancement (>50% of affected joint area)] was used (9, 12). All FOI findings were evaluated blinded to the patient group by three readers (AMG, SO, and JB) on consensus agreement. To create an optimal comparability of the FOI results of the baseline and FU study, the FOI data of the baseline study were again evaluated according to the mentioned definition.

### Statistical Analysis

Statistical analysis and data management were performed using STATA 12 (StataCorp LLC, TX, United States). The analysis of joint involvement based on CE, GS/PDUS, and FOI at the BL and FU separately was performed for the three different groups defined above.

A joint was considered to be affected if the grading was at least one (grade ≥1). GSUS was used as the reference method to determine the absolute consistency, sensitivity, and specificity with PDUS, swollen joints, and FOI (PVM, p1–p3). Further analysis included the assessment whether a joint was newly affected in FU (date of the baseline examination: T1−, date of the FU: T2+), presented with no change or was not affected anymore (T1+, T2−). The association of newly detected affected joints regarding the change of diagnosis was evaluated by calculating the area under the curve (AUC) (25) to assess the strength of association. The *post hoc* test by Sidak was used to determine whether there were significant differences in the mean number of joints detected by the different examination methods GSUS and FOI. Agreement rates were calculated by absolute agreement (in%) and prevalence-adjusted bias-adjusted kappa for FOI, GSUS, and CE for BL and FU examination. *p* < 0.05 were considered significant.

## Results

From the 60 patients examined by Erdmann-Keding et al. at BL (15), six patients could not be contacted due to loss of contact data. A total of 30 of 54 patients contacted consented to participate in this FU study resulting in 50% successful rerecruitment rate.

They were then assigned to the three different groups:

• Diagnosed PsA after baseline assessment (group I, *n* = 10).
• Still suspected PsA (group II, *n* = 6).
• Diagnosed PsA (group III, *n* = 14).

Fluorescence optical imaging could be completed in 29 patients. One examination had to be interrupted due to orthostatic dysregulation.

### Demographic Data

Results of the demographic and clinical features of the study population are shown in Table 1. At the time of FU, systemic therapy was administered to 80% of patients from group I, 67% from group II, and 93% from group III.

**TABLE 1:** Demographic and clinical data of the study population.

	All (*n* = 30)	Diagnosed PsA after baseline assessment (*n* = 10)	Still suspected PsA (*n* = 6)	Diagnosed PsA (*n* = 14)
Female (*n*)	22	8	4	10
Age in years	57.03 ± 11.01	50.4 ± 4.9	55.3 ± 13.3	62.5 ± 10.2
Duration of psoriasis	25 ± 17.4	20.7 ± 13.8	29 ± 17.9	27 ± 19.1
Duration of joint symptoms	12.3 ± 8.9	8.5 ± 6.3	16.4 ± 12.3	13.75 ± 7.7
BMI (kg/m^2^)	30.3 ± 6.2	32.9 ± 6.4	29.1 ± 5.4	29.0 ± 5.7
PASI	2.3 ± 2.47	3.6 ± 2.9	2.5 ± 2.37	1.4 ± 1.62
NAPSI right	3.9 ± 5.5	6.7 ± 7.2	0.7 ± 1.5	3.42 ± 4.5
NAPSI left	4.9 ± 6.5	8.2 ± 7.1	1.8 ± 4.1	4.2 ± 6.1
TJC (0/28)	5.9 ± 5.7	6.9 ± 6.7	5 ± 6.4	5.6 ± 4.3
SJC (0/28)	1.8 ± 2.7	2.9 ± 3.8	1.5 ± 2.1	1.2 ± 1.6
DAS28	4.3 ± 1.2	4.8 ± 1.2	3.5 ± 1.2	4.2 ± 1.0
Erosion by MSUS	8 (27%)	0 (0%)	2 (33%)	6 (43%)
Systemic therapy	25 (83.3%)	8 (80%)	4 (67%)	13 (93%)
Current MTX medication	11 (36.7%)	5 (50%)	0 (0%)	6 (42.9%)
MTX in medical history	24 (80%)	10 (100%)	3 (50%)	11 (78.6%)
Biologicals	13 (43.3%)	4 (40%)	2 (33.3%)	7 (50%)

*Data are reported by mean ± SD or n (%). PsA, psoriatic arthritis; BMI, body mass index; PASI, Psoriasis Area and Severity Index; NAPSI, Nail Psoriasis Severity Index; TJC, tender joint count; SJC, swollen joint count; DAS28, Disease Activity Score 28; MTX, methotrexate; MSUS, musculoskeletal ultrasound.*

### Comparison of Clinical Examination, Ultrasound, and Fluorescence Optical Imaging

#### Group I–Diagnosed Psoriatic Arthritis After Baseline Assessment

**FIGURE 1:**
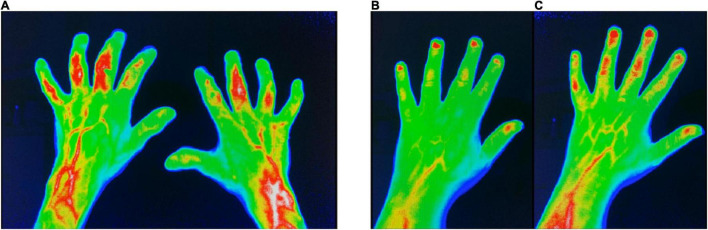
FOI in group I (change of diagnosis from suspected to confirmed PsA). **(A)** FOI in p3 with moderate signal intensities in the left PIP3 + 4 and right PIP3 + 4 joints at FU. **(B)** FOI in p2 with low signal intensities in PIP 3–5 at baseline. **(C)** FOI in p2 with moderate signal intensities in PIP 3–5 and DIP 2–4 at FU. FOI, fluorescence optical imaging; p1–p3, FOI phases 1–3; PIP, proximal interphalangeal joint; DIP, distal interphalangeal joint.

Compared to BL, patients with a change from suspected to diagnosed PsA showed an increased prevalence of joints with pathological enhancement in FOI (*p* = 0.046), especially in p2 (*p* = 0.037), and an unchanged joint distribution pattern, that is, with a dominant involvement of the DIP joints (Tables 2, 3 and Figure 1).

**TABLE 2:** Prevalence of joints with pathological findings.

		Group I *n* = 130		Group II *n* = 78		Group III *n* = 182	
CE	BL	33 (25.4%)	*p* = 0.35	4 (5.1%)	*p* = 0.15	28 (10.6%)	*p* = 0.76
	FU	44 (33.8%)		15 (19.2%)		53 (14.1%)	
GSUS	BL	15 (11.5%)	*p* = 0.005*	8 (10.3%)	*p* = 0.027*	44 (16.7%)	*p* = 0.001*
	FU	95 (73%)		34 (43.6%)		141 (37.5%)	
PDUS	BL	6 (4.6%)	*p* = 0.006*	3 (3.8%)	*p* = 0.31	8 (3.0%)	*p* = 0.004*
	FU	23 (17.7%)		5 (6.4%)		23 (6.2%)	
FOI any phase	BL	60 (46%)	*p* = 0.046*	41 (52.6%)	*p* = n.a.	184 (69.7%)	*p* = n.a.
	FU	115 (88.5%)		36 (46.2%)		159 (42.3%)	
PVM	BL	24 (40%)	*p* = 0.1	14 (34.2%)	*p* = 0.51	58 (31.5%)	*p* = 0.72
	FU	47 (40.9%)		12 (33.3%)		51 (32.1%)	
p1	BL	7 (11.7%)	*p* = 1.0	10 (24.4%)	*p* = 0.15	22 (12.0%)	*p* = 0.06
	FU	4 (3.5%)		5 (13.9%)		7 (4.4%)	
p2	BL	28 (46.7%)	*p* = 0.037*	16 (39%)	*p* = 0.74	89 (48.4%)	*p* = 0.82
	FU	55 (47.8%)		17 (47%)		89 (56.0%)	
p3	BL	1 (1.7%)	*p* = 0.26	1 (2.4%)	*p* = 0.56	15 (8.2%)	*p* = 0.55
	FU	8 (7.0%)		2 (5.6%)		12 (7.6%)	

Percentages in FOI PVM, p1, p2, p3 refer to all joints affected in FOI. The percentages in CE, GSUS, and PDUS refer to all joints considered in this group, *p ≤ 0.05. n = Number of wrist and joints examined in this group; group I, diagnosed PsA after baseline assessment; group II, still suspected PsA; group III, diagnosed PsA; BL, baseline; FU, follow-up; CE, clinical examination; GSUS, ultrasound in grayscale mode; PDUS, ultrasound in power Doppler mode; FOI, fluorescence optical imaging; p1–p3, FOI phases 1–3. n.a. = not available.

**TABLE 3:** Pattern of joint involvement according to all examination methods (CE, GSUS, and FOI).

		Group I	Group II	Group III
		*n* = 130	*n* = 78	*n* = 182
Wrist	BL	27 (23.5%)	14 (25%)	41 (15.5%)
	FU	17 (6.1%)	12 (13.3%)	30 (7.9%)
MCP	BL	17 (14.8%)	7 (12.5%)	46 (17.4%)
	FU	44 (15.9%)	27 (30.0%)	80 (21.3%)
PIP	BL	57 (49.6%)	26 (46.4%)	103 (39.0%)
	FU	114 (41.2%)	28 (31%)	140 (37.2%)
DIP	BL	17 (14.8%)	8 (14.28%)	74 (28%)
	FU	104 (37.5%)	21 (23.3%)	126 (33.5%)

*N = Number of wrists and finger joints examined in this group; group I, diagnosed PsA after baseline assessment; group II, still suspected PsA; group III, diagnosed PsA; BL, baseline; FU, follow-up; MCP, metacarpophalangeal joint; PIP, proximal interphalangeal joint; DIP, distal interphalangeal joint.*

Patients of this group were three times more common to show enhanced signal in p3 in FOI at FU – compared to BL (*p* = n.s.) (Figure 1).

In FOI, the largest number of increased signal intensity was found in FOI p2–both in 2011 and 2014 (46.7% in BL, 47.8% in FU, Table 2).

In 64.4% of pathologic joints across all examination methods, the PIP and DIP joints were affected, which increased to 78.7% in FU examination.

Significantly, more joints were affected in GSUS at FU compared to baseline examination (*p* = 0.005).

#### Group II – Suspected Psoriatic Arthritis

At FU, FOI showed a comparable number of affected joints in the group of suspected PsA (52.6% in BL, 46.2% in FU, see Table 2).

The distribution of the changes seen over the 3 phases was similar. However, in 2014, only half as many joints were detected in p1 as in 2011 (24.4% vs. 13.9%; *p* = n.s.).

At both study points, only a minimal number of increased signal intensities were found in p3 (2.4% in BL, 5.6% in FU, see Table 2).

At FU, no typical joint involvement pattern could be identified. A similar distribution of the affected finger joints with MCP (30%), PIP (31%), and DIP (23.3%) was found, with the wrists being slightly less affected (13.3%, Table 3).

Also in this group, the FU examination showed a significantly increased prevalence of affected joints by GSUS (*p* = 0.027, see Table 2).

#### Group III–Diagnosed Psoriatic Arthritis

Ultrasound in grayscale mode in the FU examination detected a significantly increased the prevalence of affected joints (*p* = 0.001), whereas the prevalence of affected joints in FOI was lower (BL: 69.7% vs. FU: 42.3%; *p* = n.s.).

This cohort showed the highest prevalence of signal enhancement in FOI phase 3, compared to the other groups (8.2% in BL, 7.6% in FU, see Table 2).

The involvement of PIP and DIP joints was mainly detected in p2 showing increased signal intensities in 80.0 and 94.3% of DIP joints in 2011 (BL) and 2014 (FU), respectively. Accordingly, the rate of affected PIP joints in p2 was 70.6% in 2011 and 84.6% in 2014 (data not shown).

Both the BL and the FU examination showed a typical pattern of joint involvement with accentuated affection of PIP (BL: 39%, FU: 37.2%) and DIP joints (BL: 28%, FU: 33.5%) (Table 3).

#### Association of Detected Newly Affected Joints by Musculoskeletal Ultrasound (GSUS/PDUS) and Fluorescence Optical Imaging in Patients in Group I Compared to Group II

Musculoskeletal ultrasound and FOI (PVM, p2) were associated with the detection of newly affected joints at FU (FOI: AUC 0.78; GSUS: AUC 0.77) more likely in group I compared to group II. More in detail, the GSUS examination method showed acceptable AUC for the PIP (PIPIII 0.72; PIPV 0.77), respectively. FOI in PVM demonstrated a similar AUC for DIPIV (0.78) and PIPII (0.72). Also, for FOI in p2, acceptable AUC in the DIPs could be determined (0.78–0.79) (see Table 4).

**TABLE 4:** Association of GSUS, PDUS, and FOI with newly suspected joints in FU with regard to the change of diagnosis (group I).

	MCP				PIP				DIP			
	II	III	IV	V	II	III	IV	V	II	III	IV	V
GSUS	0.52	0.72	0.70	0.70	0.68	0.72	0.67	0.77	0.60	0.62	0.65	0.62
PDUS	0.43	0.50	0.50	0.45	0.65	0.55	0.55	0.55	0.72	0.60	0.55	0.60
FOI PVM	0.50	0.50	0.50	0.56	0.72	0.64	0.64	0.58	0.67	0.69	0.78	0.58
FOI p1	0.50	0.50	0.50	0.50	0.56	0.61	0.56	0.50	0.56	0.50	0.50	0.50
FOI p2	0.50	0.50	0.33	0.47	0.58	0.72	0.58	0.67	0.78	0.79	0.69	0.75
FOI p3	0.50	0.50	0.50	0.50	0.56	0.61	0.61	0.56	0.50	0.56	0.50	0.42

*AUC, area under the curve; GSUS, ultrasound in grayscale mode; PDUS, ultrasound in power Doppler mode; FOI, fluorescence optical imaging; PVM, PrimaVista Mode; p1–p3, FOI phases 1–3; MCP, metacarpophalangeal joint; PIP, proximal interphalangeal joint; DIP, distal interphalangeal joint.*

#### Differences Between Baseline and Follow-Up in the Mean Number of Affected Joints Detected by the Different Methods

The mean number of joints detected as affected (≥1) differed significantly between the three groups for FOI in p2 at BL (*p* = 0.013) and FU (*p* = 0.013). The *post hoc* test by the Sidak method resulted in a significant difference between group I and group III at baseline (*p* = 0.028) and the groups I and II at the FU (*p* = 0.010). Regarding GSUS examination method, we also found a significant difference in the number of affected joints between the three groups at the time of FU (*p* = 0.003). The *post hoc* test showed a significant difference between the groups I and II (*p* = 0.002) and groups II and III (*p* = 0.013).

#### Agreement Rates of Fluorescence Optical Imaging With GSUS and Clinical Examination

Both in 2011 and 2014, agreement of CE (swollen joints) and FOI was good to very good in groups II and III (see Table 5). In group III, agreement rates in 2011 ranged from 52.8 to 88.8%, with highest accordance found in p1 and p3. Also in 2014, the agreement of CE and FOI was highest in p1 (92.9%) and p3 (88.5%). In group II, agreement rates in 2011 ranged from 80.8 to 100% and from 75.6 to 92.3% in 2014, respectively. In this group, highest agreement rates were found in the MCP and DIP joints depending on the individual phases of FOI with highest agreement found in p3 where mostly negative results were present. Agreement rates in group I extended from 71.2 to 95% at BL and from 52.9 to 89.7% at FU with highest rates for p1 and p3.

**TABLE 5:** Agreement rates (%, prevalence-adjusted bias-adjusted kappa) of FOI and GSUS (GSUS as standard of reference) vs. CE; FOI and GSUS (CE as standard of reference) for BL and FU examination.

		Group I				Group II				Group III			
		BL	kappa	FU	kappa	BL	kappa	FU	kappa	BL	kappa	FU	kappa
GSUS + FOI	PVM	79.5	0.59	49.6	–0.05	74.4	0.48	61.5	0.10	62.6	0.26	38.5	–0.22
	p1	83.8	0.68	27.3	–0.53	76.7	0.53	55.1	0.03	71.4	0.43	25.3	–0.48
	p2	76.1	0.52	49.6	0.01	74.4	0.48	55.1	–0.03	56.2	0.15	49.4	0.02
	p3	87.2	0.74	28.2	–0.48	91.0	0.81	58.9	0.00	69.8	0.40	23.6	–0.50
CE + FOI	PVM	75.9	0.52	56.4	0.13	82.7	0.65	82.0	0.64	66.5	0.33	72.5	0.45
	p1	91.3	0.83	88.9	0.78	80.8	0.62	91.0	0.82	85.2	0.70	92.9	0.85
	p2	71.2	0.42	52.9	0.06	80.8	0.62	75.6	0.51	52.8	0.05	53.3	0.08
	p3	95.2	0.90	89.7	0.79	100	1	92.3	0.85	88.8	0.74	88.5	0.77
CE + GSUS		85.6	0.71	30	–0.24	92.3	0.88	53.8	0.01	76.4	0.52	26.4	–0.42

*Group I, diagnosed PsA after baseline assessment; group II, still suspected PsA; group III, diagnosed PsA; BL, baseline; FU, follow-up; CE, clinical examination; FOI, fluorescence optical imaging; PVM, FOI PrimaVista Mode; p1–p3, FOI phases 1–3; GSUS, ultrasound in grayscale mode.*

In all 3 groups, the agreement of FOI and GSUS was better at BL than at FU, which depends on the individual phases of FOI. In group III, agreement rates of GSUS and FOI ranged from 56.2 to 71.4% in 2011 and from 23.6 to 49.4% in 2014, respectively. Corresponding agreement rates in group II ranged from 74.4 to 91% in 2011, with the highest agreements found in p3, and from 55.1 to 61.5% in FU. Also in group I, GSUS and FOI showed lower agreement rates ranging from 27.3 to 49.6% in 2014 with p2 and PVM exhibiting the highest agreement rates. The best agreement rate was found for PIPV (p2 and PVM) with 88.9%.

##### Safety

No side effects to the FOI examination or to indocyanine green (ICG) were detected during the study.

## Discussion

As far as we know, this is the first study presenting FU data on FOI results in patients with PsA or rather early PsA. Since PsA – as chronic, progressive disease in the majority of patients–results in radiological damage in up to 47% of patients at a median interval of 2 years (26), there is a great need for an objective and sensitive screening tool. Thus, the aim of this study was to explore the value of FOI to distinguish between acute and chronic disease stages for screening purpose.

We found that newly detected joints by MSUS and FOI (PVM, p2) in FU were positively associated with the change of diagnosis from suspected to confirmed PsA. These results match the findings of significantly increased number of joints with pathological findings in group I in FU, with the DIP joints being particularly affected.

The number of joints detected as affected in FOI p2 at the two study points differed significantly between the three groups (*p* = 0.013 at BL and FU) and between groups I and II at FU (*p* = 0.010). This indicates that FOI is able to distinguish between patients with clear and suspected PsA. Phase 2 seems to be most sensitive for this purpose, which underlines the importance of this phase for subclinical inflammation, as described previously (12).

Recent studies assumed that FOI p3 shows increased capillary permeability in which the dye ICG (indocyanine green) is more persistent than normal, which represents chronic changes that only develop in the course of disease (10, 27). This is consistent with our finding that patients with diagnosed PsA showed the highest prevalence of signal enhancement in FOI p3. Correspondingly, we found erosions in 43% of this group in the GSUS. Unlike group II with unchanged suspected PsA where almost no changes could be found in FOI p3, signal enhancement in p3 was three times more frequent than at the time of the baseline study in group I. Interestingly, the signal enhancements found at FU in this phase were mainly slight changes (grade 1). In case that only higher-grade changes (grade ≥2) had been considered as an evaluation criterion, this information would have been lost, since only one joint in this cohort showed a higher-grade change in FOI p3 in the FU. This could be possibly taken into account for diagnostic and consequently further therapeutic decisions.

Comparable studies (10, 12) already described that especially the flooding in and the washing out of ICG may depend on an increased and dysregulated microcirculation, which leads to the assumption that phase 1 visualizes active inflammation. In contrast to Erdmann-Keding et al. (15), the group with unchanged suspected PsA did not show higher-grade changes in FOI p1. However, this may be explained by a falsification of the results due to a systematic therapy existing at the time of FU in these patients. Glimm et al. detected statistically significant reduction in FOI sum score (FOIAS – fluorescence optical imaging activity score) when they investigated FOI as a tool for therapy monitoring in patients with early and active rheumatoid arthritis (RA) under DMARD therapy in a 1-year FU period (10). Therefore, it is possible that our study may have found fewer changes in FOI phase 1, since 67% of the patients with unchanged suspected PsA already received systematic therapy at the time of FU (whereas only 29% received csDMARDs (conventional synthetical) and 23% bDMARDs (biologic) at baseline).

We found good to very good association of FOI and CE (swollen joints) with FOI p1 and p3, which shows the highest accordance for the groups II and III. This result is consistent with the findings of Werner et al., which showed highest agreement between FOI p1 and swollen and tender joints indicating that p1 displays joints with high clinical activity (9). The good agreement of FOI p3 and CE in group III can be explained by already existing chronic joint changes, which are reflected in the FOI in p3 as chronic capillary leakage. In group II, there were almost no changes in FOI p3 in clinically unaffected joints. This is in line with the findings of a systematic literature review (SLR) by Zabotti et al. presenting that the risk of PsA development in PsO patients with arthralgia was about two times greater than in subjects without arthralgia (28).

Disagreement of FOI p2 and CE results from a higher rate of positive findings in FOI (see also Supplementary Data and Supplementary Table 1). This may underline the importance of this phase for subclinical inflammation, which cannot yet be detected by clinical investigation. It is also consistent with the findings of Werner et al. who found positive findings in FOI in 45% of clinically asymptomatic joints (9). The visualization of changes in microcirculation and vascularization by FOI may enable the detection of a very early PsA disease state in a pre–subclinical phase – in transition to a clinical stage (29). This is underlined by the hypothesis that non-specific musculoskeletal symptoms in patients with psoriasis may actually represent a preclinical phase of PsA (30, 31). In addition, Faustini et al. reported that the risk for developing PsA was as high as 60% if patients had subclinical synovitis and symptoms related to arthralgia highlighting the importance to establish an early PsA diagnosis (32).

Unlike ultrasound, FOI examination can be performed by medical assistants and does not require the presence of a medical physician. Although the analysis must be performed by a physician, it can be undertaken at a different time and thus offers flexibility. Even if the injection of ICG is necessary for the procedure of FOI, it has been shown that ICG is well tolerated and side effects (i.e., anaphylactic reaction) occur rarely (1:42,000) (33). FOI can therefore be regarded as a safe examination method and is also associated with a low expenditure of time. All these advantages of the FOI method allow an easy integration of the examination into the clinical routine.

Study limitations include the relatively low number of patients in this FU study. To further investigate FOI’s ability to detect early PsA, larger-scale studies with several FU examinations are necessary. This study characterizes a pilot study to (further) explore the role of FOI as a screening tool for PsA development. Furthermore, patients of all three groups were under systemic therapy at the time of the FU, which implies that our findings might not reflect the “natural state” of disease. However, of the patients from group II who received systemic therapy at the time of FU, one-third received it exclusively for psoriatic skin lesions and another one-third for symptomatic joint pain. A further limitation of the study might have arisen from a possible interference of the results by osteoarthritis (OA), which might have led to false-positive results due to the inflammatory effect of (early) OA.

This work supports the findings of the baseline study and therefore provides further evidence that FOI is able to distinguish between acute and chronic disease stages. Hence, FOI can be considered as an useful screening tool for the early diagnosis of PsA. Since a delay in diagnosis impacts on long-term joint damage and functional disability (4, 5), its application in daily routine can help to diagnose early PsA in time to prevent progressive joint damage. An integration of this method as screening for prompt recognition of patients demanding a further referral can contribute to achieve this goal.

## Data Availability Statement

The raw data supporting the conclusions of this article will be made available by the authors, without undue reservation.

## Ethics Statement

The studies involving human participants were reviewed and approved by the Local Medical Ethical Committee of the Charité – Universitätsmedizin, Berlin, Germany. All patients provided informed consent to participate in the study. The patients/participants provided their written informed consent to participate in this study.

## Author Contributions

JB, A-MG, JK, and SO made substantial contributions to the conception and design of the work, the acquisition, analysis, and interpretation of data for the work. JB, A-MG, GK, ME-K, G-RB, PH, JK, and SO were drafting the work and revising it critically for important intellectual content. All authors provide approval for publication of the content and agreed to be accountable for all aspects of the work in ensuring that questions related to the accuracy and integrity of any part of the work are appropriately investigated and resolved.

## Conflict of Interest

The authors declare that the research was conducted in the absence of any commercial or financial relationships that could be construed as a potential conflict of interest.

## Publisher’s Note

All claims expressed in this article are solely those of the authors and do not necessarily represent those of their affiliated organizations, or those of the publisher, the editors and the reviewers. Any product that may be evaluated in this article, or claim that may be made by its manufacturer, is not guaranteed or endorsed by the publisher.

## Abbreviations

AUC: area under the curve
bDMARDs: biologic disease modifying antirheumatic drugs
BL: baseline data baseline study
BMI: body mass index
BSA: body surface area
CASPAR: ClASsification criteria for Psoriatic Arthritis
CE: clinical examination
csDMARDs: conventional synthetical disease modifying antirheumatic drugs
DAS28: Disease Activity Score of 28 joints
DIP: distal interphalangeal joints II–V of the fingers
DMARD: Disease modifying antirheumatic Drugs
EULAR: European League Against Rheumatism
FOI: fluorescence optical imaging
FOIAS: fluorescence optical imaging activity score
FU: follow-up study
GS(US): grayscale mode grayscale ultrasound
ICG: indocyanine green
MCP: metacarpophalangeal joints I–V of the fingers
MRI: magnetic resonance imaging
MSUS: musculoskeletal ultrasound
MTX: methotrexate
NAPSI: nail psoriasis severity index
OA: osteoarthritis
OMERACT: outcome measures in rheumatology
PASI: Psoriasis Area and Severity Index (PASI)
PD(US): power Doppler mode power Doppler ultrasound
PIP: proximal interphalangeal joints II–V of the fingers
PsA: psoriatic arthritis
PsO: psoriasis
PVM: PrimaVista Mode in FOI
p1: phase 1 in FOI
p2: phase 2 in FOI
p3: phase 3 in FOI
RA: rheumatoid arthritis
SJC: swollen joint count
TJC: tender joint count
T1: date of baseline examination
T2: date of follow-up examination
VAS: visual analog scale.
