# Supplementary material for: Follow-Up Comparison of Fluorescence Optical Imaging With Musculoskeletal Ultrasound for Early Detection of Psoriatic Arthritis
Source: Front Med (Lausanne). 2022 Mar 18;9:845545. doi: 10.3389/fmed.2022.845545 (PMC8971374; doi:10.3389/fmed.2022.845545)
Supplement: Supplementary file 1 [file Data_Sheet_1.docx]

Supplementary data

**Sensitivity and specificity of FOI with GSUS as reference method**

**Group I-III:**

Using MSUS in greyscale as reference, inflammatory changes in the joints were diagnosed in all three cohorts by means of FOI in p1 and p3 with high specificity and low sensitivity.

In all three cohorts, the specificity for p1 was between 84.1% and 88.3% at BL and between 91.7% and 98.1% at FU. Corresponding specificity for p3 for all 3 cohorts was between 89.8% and 100% in 2011 and 89.6% and 100% in 2014. However, corresponding sensitivities for p1 and p3 seen in all three cohorts were very low, ranging from 0% to 13% in 2011 and 3.1% to 9.2% in 2014. For p2, the sensitivities of the three cohorts were slightly higher ranging from 22.2% to 47.8% in 2011 and 35.5% to 49.7% in 2014, with group II showing the lowest sensitivities of all three cohorts (Table S3).

**Differences in MSUS ≥ grade 2 between the three defined groups**

Even if a joint is defined as affected from grade ≥2, significantly more joints were detected in GSUS in group I and III at follow-up (p=0.007 and p=0.002).

In group III, it is noticeable that in the baseline as well as in the follow-up examination, higher grade joint changes were mainly found in the MCP joints (50% BL, 40% FU).

In group II, only a few joints with grade ≥2 could be detected at both study points in GSUS (2.6% in BL, 10.3% in FU), these were mainly found in the wrists (50% in BL, 37.5% in FU). In group I, only slight changes (grade 1) in the PIP and DIP joints were found at BL in GSUS. In FU, however, 30% of the GSUS-detected joints with grade ≥2 were found in the PIP joints and 30% in the DIP joints.

**Differences in FOI** **≥ grade 2 between the three defined groups**

If a joint in the FOI was evaluated as affected from grade ≥2, similar results were found in all three groups compared to a rating from grade ≥1. However, fewer joints in p3 were detected in group III at FU according to this evaluation criterion (1.5% vs. 7.6%). Furthermore, it could not be confirmed that patients in group I were three times more common to show signal enhancement in p3 during follow-up: at BL, no joint with grade ≥2 could be detected in the FOI in p3, at FU only one single joint was detected.

**Table S1:** Sensitivity (%) and specificity (%) of FOI and CE with GSUS as standard of reference.

|  |  | Group I | | Group II | | Group III | |
| --- | --- | --- | --- | --- | --- | --- | --- |
|  |  | Sensitivity | Specificity | Sensitivity | Specificity | Sensitivity | Specificity |
| CE | BL | 16.7 | 94.2 | 0 | 100 | 9.7 | 97.3 |
|  | FU | 8.1 | 85.6 | 3.9 | 94.4 | 5.9 | 98.1 |
| FOI (PVM) | BL | 40.5 | 82.4 | 5.6 | 80.8 | 25.6 | 69.2 |
|  | FU | 41.4 | 65.4 | 33.2 | 93.5 | 28.4 | 79.5 |
| FOI (p1) | BL | 2.4 | 92.8 | 0 | 84.1 | 13.0 | 88.3 |
|  | FU | 4.6 | 91.7 | 3.1 | 95.0 | 4,3 | 98.1 |
| FOI (p2) | BL | 40.5 | 80.4 | 22.2 | 77.7 | 47.8 | 53.6 |
|  | FU | 45.4 | 56.3 | 35.5 | 82.7 | 49.7 | 58.9 |
| FOI (p3) | BL | 0 | 98.9 | 5.55 | 100 | 2.4 | 89.8 |
|  | FU | 6.2 | 89.6 | 9.2 | 100 | 5.2 | 90.4 |

**Table S1:** Sensitivity (%) and specificity (%) of FOI and CE (GSUS as standard of reference).

*Group I*: Diagnosed PsA after baseline assessment, *group II*: still suspected PsA, *group III*: diagnosed PsA, *BL:* Baseline, *FU:* Follow up, *CE*: Clinical examination, *FOI*: fluorescence optical imaging, *PVM*: FOI Prima Vista Mode, *p1-p3:* FOI phases 1-3.

**Figure S1.** Distribution of patients at Baseline (BL) and Follow-up (FU)

Number of patients

**Figure S1:** Distribution of patients at Baseline (BL) and Follow-up (FU):

Diagnosed PsA after baseline assessment (Group I), Still suspected PsA (Group II), (Unchanged) Diagnosed PsA (Group III)
